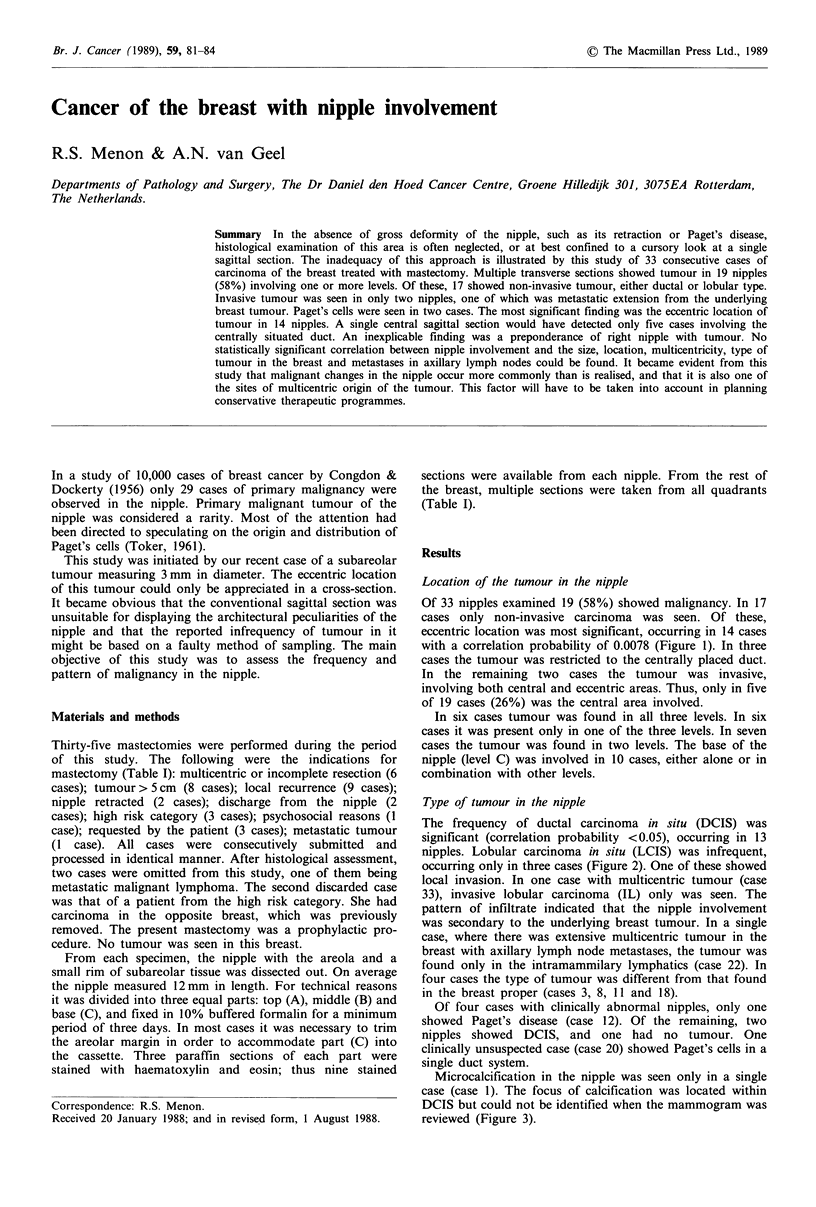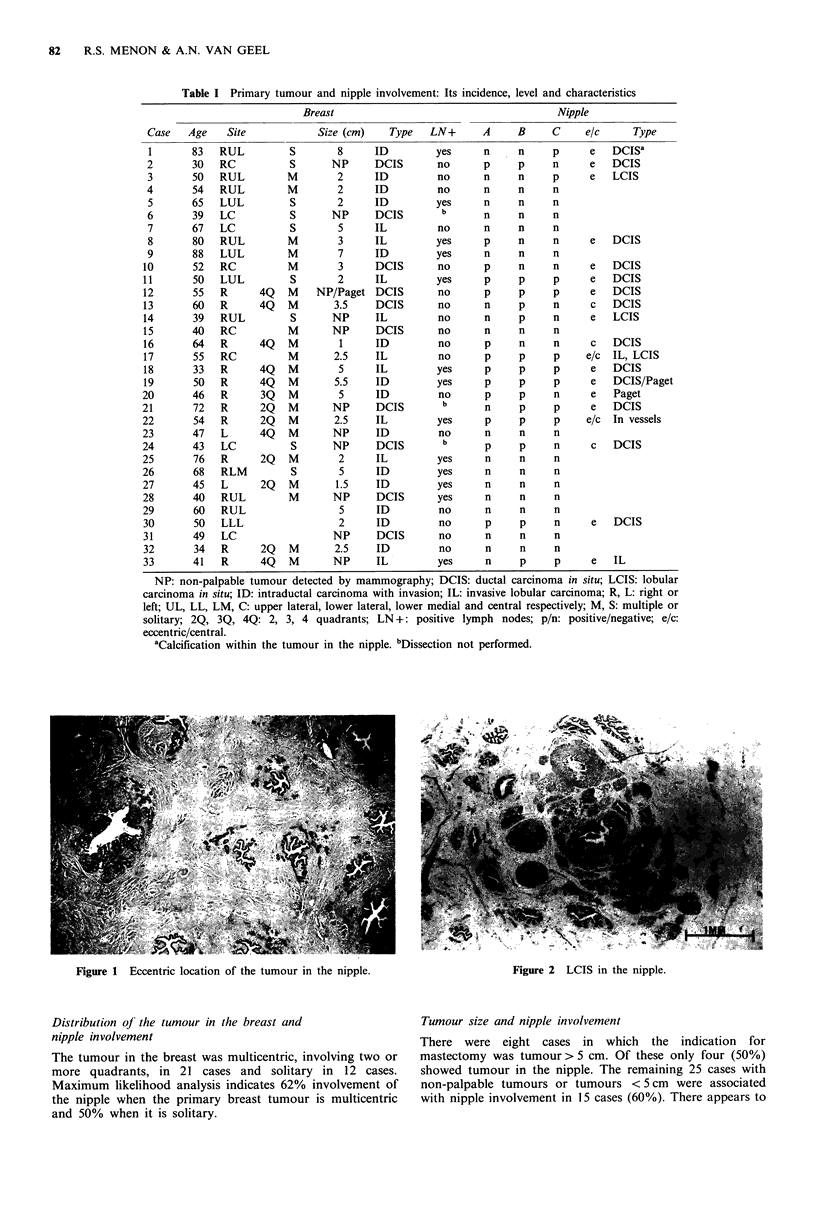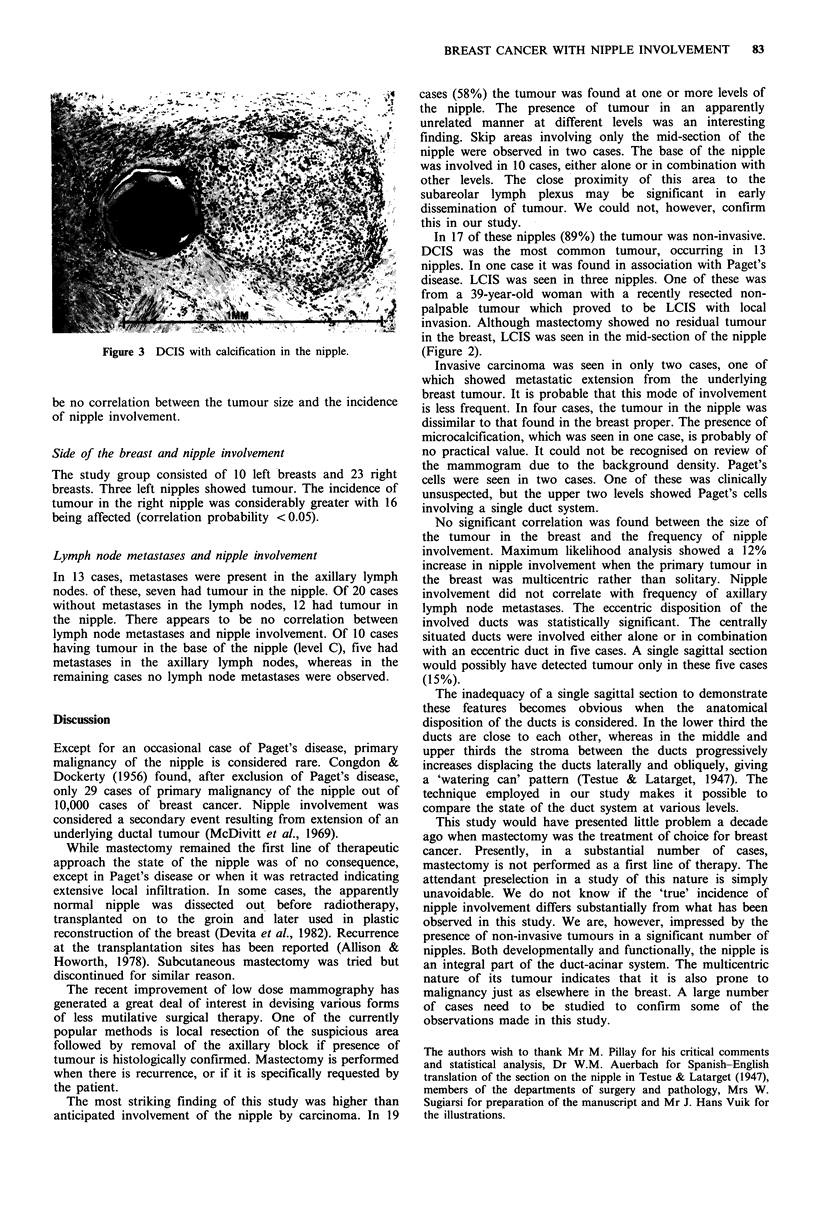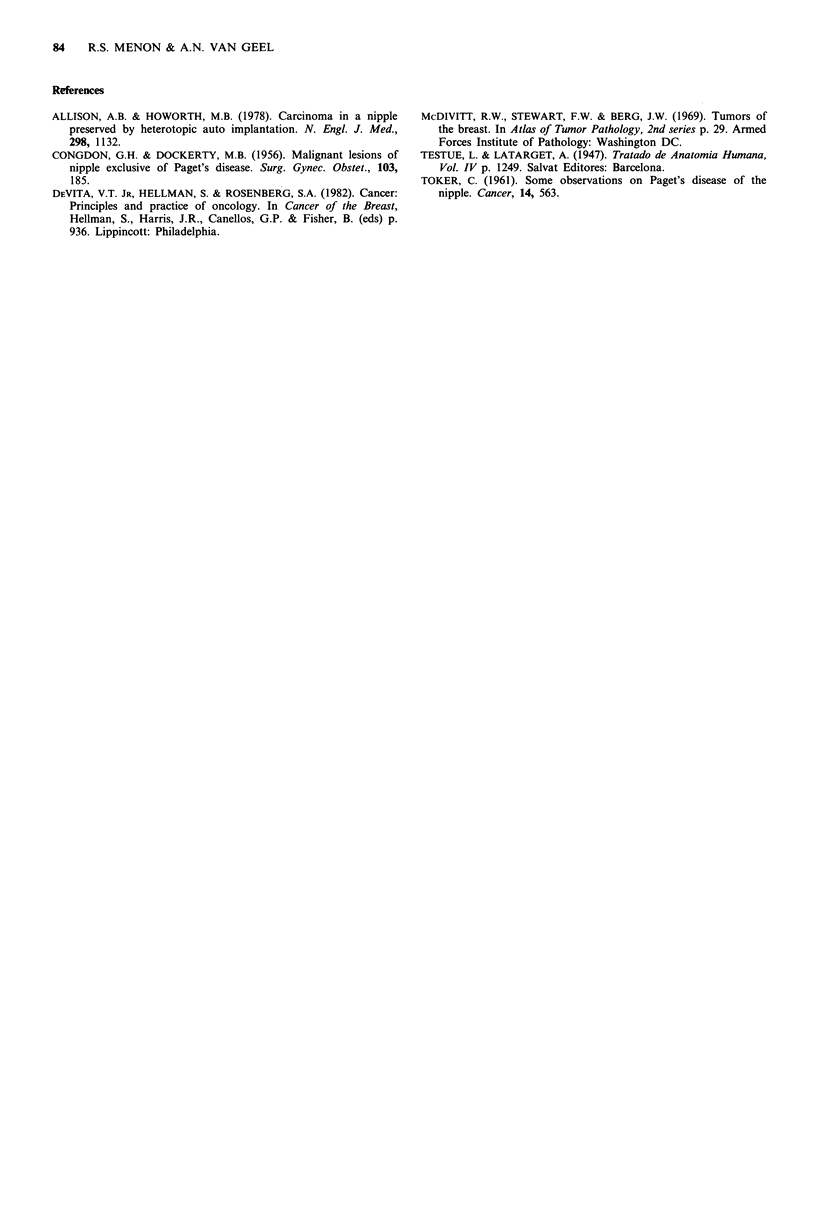# Cancer of the breast with nipple involvement.

**DOI:** 10.1038/bjc.1989.15

**Published:** 1989-01

**Authors:** R. S. Menon, A. N. van Geel

**Affiliations:** Department of Pathology Dr Daniel den Hoed Cancer Centre, Rotterdam, The Netherlands.

## Abstract

**Images:**


Br. J. Cancer (1989), 59, 81–84 © The Macmillan Press Ltd., 1989

### Cancer of the breast with nipple involvement

R.S. Menon & A.N. van Geel

*Departments of Pathology and Surgery, The Dr Daniel den Hoed Cancer Centre, Groene Hilledijk 301, 3075EA Rotterdam, The Netherlands.*

**Summary** In the absence of gross deformity of the nipple, such as its retraction or Paget's disease, histological examination of this area is often neglected, or at best confined to a cursory look at a single sagittal section. The inadequacy of this approach is illustrated by this study of 33 consecutive cases of carcinoma of the breast treated with mastectomy. Multiple transverse sections showed tumour in 19 nipples (58%) involving one or more levels. Of these, 17 showed non-invasive tumour, either ductal or lobular type. Invasive tumour was seen in only two nipples, one of which was metastatic extension from the underlying breast tumour. Paget's cells were seen in two cases. The most significant finding was the eccentric location of tumour in 14 nipples. A single central sagittal section would have detected only five cases involving the centrally situated duct. An inexplicable finding was a preponderance of right nipple with tumour. No statistically significant correlation between nipple involvement and the size, location, multicentricity, type of tumour in the breast and metastases in axillary lymph nodes could be found. It became evident from this study that malignant changes in the nipple occur more commonly than is realised, and that it is also one of the sites of multicentric origin of the tumour. This factor will have to be taken into account in planning conservative therapeutic programmes.

In a study of 10,000 cases of breast cancer by Congdon & Dockerty (1956) only 29 cases of primary malignancy were observed in the nipple. Primary malignant tumour of the nipple was considered a rarity. Most of the attention had been directed to speculating on the origin and distribution of Paget's cells (Toker, 1961).

This study was initiated by our recent case of a subareolar tumour measuring 3 mm in diameter. The eccentric location of this tumour could only be appreciated in a cross-section. It became obvious that the conventional sagittal section was unsuitable for displaying the architectural peculiarities of the nipple and that the reported infrequency of tumour in it might be based on a faulty method of sampling. The main objective of this study was to assess the frequency and pattern of malignancy in the nipple.

### Materials and methods

Thirty-five mastectomies were performed during the period of this study. The following were the indications for mastectomy (Table I): multicentric or incomplete resection (6 cases); tumour > 5 cm (8 cases); local recurrence (9 cases); nipple retracted (2 cases); discharge from the nipple (2 cases); high risk category (3 cases); psychosocial reasons (1 case); requested by the patient (3 cases); metastatic tumour (1 case). All cases were consecutively submitted and processed in identical manner. After histological assessment, two cases were omitted from this study, one of them being metastatic malignant lymphoma. The second discarded case was that of a patient from the high risk category. She had carcinoma in the opposite breast, which was previously removed. The present mastectomy was a prophylactic procedure. No tumour was seen in this breast.

From each specimen, the nipple with the areola and a small rim of subareolar tissue was dissected out. On average the nipple measured 12 mm in length. For technical reasons it was divided into three equal parts: top (A), middle (B) and base (C), and fixed in 10% buffered formalin for a minimum period of three days. In most cases it was necessary to trim the areolar margin in order to accommodate part (C) into the cassette. Three paraffin sections of each part were stained with haematoxylin and eosin; thus nine stained sections were available from each nipple. From the rest of the breast, multiple sections were taken from all quadrants (Table I).

Correspondence: R.S. Menon.
Received 20 January 1988; and in revised form, 1 August 1988.

### Results

*Location of the tumour in the nipple*

Of 33 nipples examined 19 (58%) showed malignancy. In 17 cases only non-invasive carcinoma was seen. Of these, eccentric location was most significant, occurring in 14 cases with a correlation probability of 0.0078 (Figure 1). In three cases the tumour was restricted to the centrally placed duct. In the remaining two cases the tumour was invasive, involving both central and eccentric areas. Thus, only in five of 19 cases (26%) was the central area involved.

In six cases tumour was found in all three levels. In six cases it was present only in one of the three levels. In seven cases the tumour was found in two levels. The base of the nipple (level C) was involved in 10 cases, either alone or in combination with other levels.

*Type of tumour in the nipple*

The frequency of ductal carcinoma *in situ* (DCIS) was significant (correlation probability $<0.05$), occurring in 13 nipples. Lobular carcinoma *in situ* (LCIS) was infrequent, occurring only in three cases (Figure 2). One of these showed local invasion. In one case with multicentric tumour (case 33), invasive lobular carcinoma (IL) only was seen. The pattern of infiltrate indicated that the nipple involvement was secondary to the underlying breast tumour. In a single case, where there was extensive multicentric tumour in the breast with axillary lymph node metastases, the tumour was found only in the intramammilary lymphatics (case 22). In four cases the type of tumour was different from that found in the breast proper (cases 3, 8, 11 and 18).

Of four cases with clinically abnormal nipples, only one showed Paget's disease (case 12). Of the remaining, two nipples showed DCIS, and one had no tumour. One clinically unsuspected case (case 20) showed Paget's cells in a single duct system.

Microcalcification in the nipple was seen only in a single case (case 1). The focus of calcification was located within DCIS but could not be identified when the mammogram was reviewed (Figure 3).

**Table I** Primary tumour and nipple involvement: Its incidence, level and characteristics

| | *Breast* | | | | | | | *Nipple* | | | | |
|---|---|---|---|---|---|---|---|---|---|---|---|---|
| *Case* | *Age* | *Site* | | | *Size (cm)* | *Type* | *LN+* | *A* | *B* | *C* | *e/c* | *Type* |
| 1 | 83 | RUL | | S | 8 | ID | yes | n | n | p | e | DCIS[a] |
| 2 | 30 | RC | | S | NP | DCIS | no | p | p | n | e | DCIS |
| 3 | 50 | RUL | | M | 2 | ID | no | n | n | p | e | LCIS |
| 4 | 54 | RUL | | M | 2 | ID | no | n | n | n | | |
| 5 | 65 | LUL | | S | 2 | ID | yes | n | n | n | | |
| 6 | 39 | LC | | S | NP | DCIS | [b] | n | n | n | | |
| 7 | 67 | LC | | S | 5 | IL | no | n | n | n | | |
| 8 | 80 | RUL | | M | 3 | IL | yes | p | n | n | e | DCIS |
| 9 | 88 | LUL | | M | 7 | ID | yes | n | n | n | | |
| 10 | 52 | RC | | M | 3 | DCIS | no | p | n | n | e | DCIS |
| 11 | 50 | LUL | | S | 2 | IL | yes | p | p | p | e | DCIS |
| 12 | 55 | R | 4Q | M | NP/Paget | DCIS | no | p | p | p | e | DCIS |
| 13 | 60 | R | 4Q | M | 3.5 | DCIS | no | n | p | n | c | DCIS |
| 14 | 39 | RUL | | S | NP | IL | no | n | p | n | e | LCIS |
| 15 | 40 | RC | | M | NP | DCIS | no | n | n | n | | |
| 16 | 64 | R | 4Q | M | 1 | ID | no | p | n | n | c | DCIS |
| 17 | 55 | RC | | M | 2.5 | IL | no | p | p | p | e/c | IL, LCIS |
| 18 | 33 | R | 4Q | M | 5 | IL | yes | p | p | p | e | DCIS |
| 19 | 50 | R | 4Q | M | 5.5 | ID | yes | p | p | p | e | DCIS/Paget |
| 20 | 46 | R | 3Q | M | 5 | ID | no | p | p | n | e | Paget |
| 21 | 72 | R | 2Q | M | NP | DCIS | [b] | n | p | p | e | DCIS |
| 22 | 54 | R | 2Q | M | 2.5 | IL | yes | p | p | p | e/c | In vessels |
| 23 | 47 | L | 4Q | M | NP | ID | no | n | n | n | | |
| 24 | 43 | LC | | S | NP | DCIS | [b] | p | p | n | c | DCIS |
| 25 | 76 | R | 2Q | M | 2 | IL | yes | n | n | n | | |
| 26 | 68 | RLM | | S | 5 | ID | yes | n | n | n | | |
| 27 | 45 | L | 2Q | M | 1.5 | ID | yes | n | n | n | | |
| 28 | 40 | RUL | | M | NP | DCIS | yes | n | n | n | | |
| 29 | 60 | RUL | | | 5 | ID | no | n | n | n | | |
| 30 | 50 | LLL | | | 2 | ID | no | p | p | n | e | DCIS |
| 31 | 49 | LC | | | NP | DCIS | no | n | n | n | | |
| 32 | 34 | R | 2Q | M | 2.5 | ID | no | n | n | n | | |
| 33 | 41 | R | 4Q | M | NP | IL | yes | n | p | p | e | IL |

NP: non-palpable tumour detected by mammography; DCIS: ductal carcinoma *in situ*; LCIS: lobular carcinoma *in situ*; ID: intraductal carcinoma with invasion; IL: invasive lobular carcinoma; R, L: right or left; UL, LL, LM, C: upper lateral, lower lateral, lower medial and central respectively; M, S: multiple or solitary; 2Q, 3Q, 4Q: 2, 3, 4 quadrants; LN+: positive lymph nodes; p/n: positive/negative; e/c: eccentric/central.

[a]Calcification within the tumour in the nipple. [b]Dissection not performed.

**Figure 1** Eccentric location of the tumour in the nipple.

**Figure 2** LCIS in the nipple.

*Distribution of the tumour in the breast and nipple involvement*

The tumour in the breast was multicentric, involving two or more quadrants, in 21 cases and solitary in 12 cases. Maximum likelihood analysis indicates 62% involvement of the nipple when the primary breast tumour is multicentric and 50% when it is solitary.

*Tumour size and nipple involvement*

There were eight cases in which the indication for mastectomy was tumour > 5 cm. Of these only four (50%) showed tumour in the nipple. The remaining 25 cases with non-palpable tumours or tumours < 5 cm were associated with nipple involvement in 15 cases (60%). There appears to

**Figure 3** DCIS with calcification in the nipple.

be no correlation between the tumour size and the incidence of nipple involvement.

*Side of the breast and nipple involvement*

The study group consisted of 10 left breasts and 23 right breasts. Three left nipples showed tumour. The incidence of tumour in the right nipple was considerably greater with 16 being affected (correlation probability $<0.05$).

*Lymph node metastases and nipple involvement*

In 13 cases, metastases were present in the axillary lymph nodes. of these, seven had tumour in the nipple. Of 20 cases without metastases in the lymph nodes, 12 had tumour in the nipple. There appears to be no correlation between lymph node metastases and nipple involvement. Of 10 cases having tumour in the base of the nipple (level C), five had metastases in the axillary lymph nodes, whereas in the remaining cases no lymph node metastases were observed.

### Discussion

Except for an occasional case of Paget's disease, primary malignancy of the nipple is considered rare. Congdon & Dockerty (1956) found, after exclusion of Paget's disease, only 29 cases of primary malignancy of the nipple out of 10,000 cases of breast cancer. Nipple involvement was considered a secondary event resulting from extension of an underlying ductal tumour (McDivitt *et al.*, 1969).

While mastectomy remained the first line of therapeutic approach the state of the nipple was of no consequence, except in Paget's disease or when it was retracted indicating extensive local infiltration. In some cases, the apparently normal nipple was dissected out before radiotherapy, transplanted on to the groin and later used in plastic reconstruction of the breast (Devita *et al.*, 1982). Recurrence at the transplantation sites has been reported (Allison & Howorth, 1978). Subcutaneous mastectomy was tried but discontinued for similar reason.

The recent improvement of low dose mammography has generated a great deal of interest in devising various forms of less mutilative surgical therapy. One of the currently popular methods is local resection of the suspicious area followed by removal of the axillary block if presence of tumour is histologically confirmed. Mastectomy is performed when there is recurrence, or if it is specifically requested by the patient.

The most striking finding of this study was higher than anticipated involvement of the nipple by carcinoma. In 19 cases (58%) the tumour was found at one or more levels of the nipple. The presence of tumour in an apparently unrelated manner at different levels was an interesting finding. Skip areas involving only the mid-section of the nipple were observed in two cases. The base of the nipple was involved in 10 cases, either alone or in combination with other levels. The close proximity of this area to the subareolar lymph plexus may be significant in early dissemination of tumour. We could not, however, confirm this in our study.

In 17 of these nipples (89%) the tumour was non-invasive. DCIS was the most common tumour, occurring in 13 nipples. In one case it was found in association with Paget's disease. LCIS was seen in three nipples. One of these was from a 39-year-old woman with a recently resected non-palpable tumour which proved to be LCIS with local invasion. Although mastectomy showed no residual tumour in the breast, LCIS was seen in the mid-section of the nipple (Figure 2).

Invasive carcinoma was seen in only two cases, one of which showed metastatic extension from the underlying breast tumour. It is probable that this mode of involvement is less frequent. In four cases, the tumour in the nipple was dissimilar to that found in the breast proper. The presence of microcalcification, which was seen in one case, is probably of no practical value. It could not be recognised on review of the mammogram due to the background density. Paget's cells were seen in two cases. One of these was clinically unsuspected, but the upper two levels showed Paget's cells involving a single duct system.

No significant correlation was found between the size of the tumour in the breast and the frequency of nipple involvement. Maximum likelihood analysis showed a 12% increase in nipple involvement when the primary tumour in the breast was multicentric rather than solitary. Nipple involvement did not correlate with frequency of axillary lymph node metastases. The eccentric disposition of the involved ducts was statistically significant. The centrally situated ducts were involved either alone or in combination with an eccentric duct in five cases. A single sagittal section would possibly have detected tumour only in these five cases (15%).

The inadequacy of a single sagittal section to demonstrate these features becomes obvious when the anatomical disposition of the ducts is considered. In the lower third the ducts are close to each other, whereas in the middle and upper thirds the stroma between the ducts progressively increases displacing the ducts laterally and obliquely, giving a 'watering can' pattern (Testue & Latarget, 1947). The technique employed in our study makes it possible to compare the state of the duct system at various levels.

This study would have presented little problem a decade ago when mastectomy was the treatment of choice for breast cancer. Presently, in a substantial number of cases, mastectomy is not performed as a first line of therapy. The attendant preselection in a study of this nature is simply unavoidable. We do not know if the 'true' incidence of nipple involvement differs substantially from what has been observed in this study. We are, however, impressed by the presence of non-invasive tumours in a significant number of nipples. Both developmentally and functionally, the nipple is an integral part of the duct-acinar system. The multicentric nature of its tumour indicates that it is also prone to malignancy just as elsewhere in the breast. A large number of cases need to be studied to confirm some of the observations made in this study.

The authors wish to thank Mr M. Pillay for his critical comments and statistical analysis, Dr W.M. Auerbach for Spanish–English translation of the section on the nipple in Testue & Latarget (1947), members of the departments of surgery and pathology, Mrs W. Sugiarsi for preparation of the manuscript and Mr J. Hans Vuik for the illustrations.

### References

ALLISON, A.B. & HOWORTH, M.B. (1978). Carcinoma in a nipple preserved by heterotopic auto implantation. *N. Engl. J. Med.*, **298,** 1132.

CONGDON, G.H. & DOCKERTY, M.B. (1956). Malignant lesions of nipple exclusive of Paget's disease. *Surg. Gynec. Obstet.*, **103,** 185.

DeVITA, V.T. Jr, HELLMAN, S. & ROSENBERG, S.A. (1982). Cancer: Principles and practice of oncology. In *Cancer of the Breast*, Hellman, S., Harris, J.R., Canellos, G.P. & Fisher, B. (eds) p. 936. Lippincott: Philadelphia.

McDIVITT, R.W., STEWART, F.W. & BERG, J.W. (1969). Tumors of the breast. In *Atlas of Tumor Pathology, 2nd series* p. 29. Armed Forces Institute of Pathology: Washington DC.

TESTUE, L. & LATARGET, A. (1947). *Tratado de Anatomia Humana, Vol. IV* p. 1249. Salvat Editores: Barcelona.

TOKER, C. (1961). Some observations on Paget's disease of the nipple. *Cancer*, **14,** 563.